# Complete Genome Sequences of Bacteriophages Pulchra and Vanisius Isolated on *Microbacterium foliorum* .

**DOI:** 10.17912/micropub.biology.001860

**Published:** 2026-09-28

**Authors:** Elvira R. Eivazova, Gregory S. Markov, Tessa C. Cote Allen, Ava E. Brittain, Jenna St. Pierre, Madalyn N Ellis, Michael J. Pollack

**Affiliations:** 1 Columbia State Community College, Columbia, Tennessee, United States

## Abstract

Actinobacteriophages Pulchra and Vanisius were isolated from soil samples using *Microbacterium foliorum* NRRL B-24224. Pulchra and Vanisius have genomes of 53312 bp and 17453bp encoding 91 and 25 predicted protein-coding genes, respectively. Both phages exhibit a siphovirus morphology and have the same GC content of 68.8%. Based on gene content, Pulchra and Vanisius are assigned to actinobacteriophage clusters EC and EE, correspondingly.

**Figure 1. Transmission electron microscopy images of phages Pulchra (A) and Vanisius (B):** The TEM images show a siphovirus morphology with the characteristic icosahedral capsid and tail. A Hitachi H-7650 Transmission Electron Microscope (Tokyo, Japan) was used for bacteriophage imaging with an accelerating voltage of 100 kV. Bacteriophage samples were stained using 1% uranyl acetate on copper grids attached to Pelco Tabs (Ted Peller, Inc., Redding, CA).

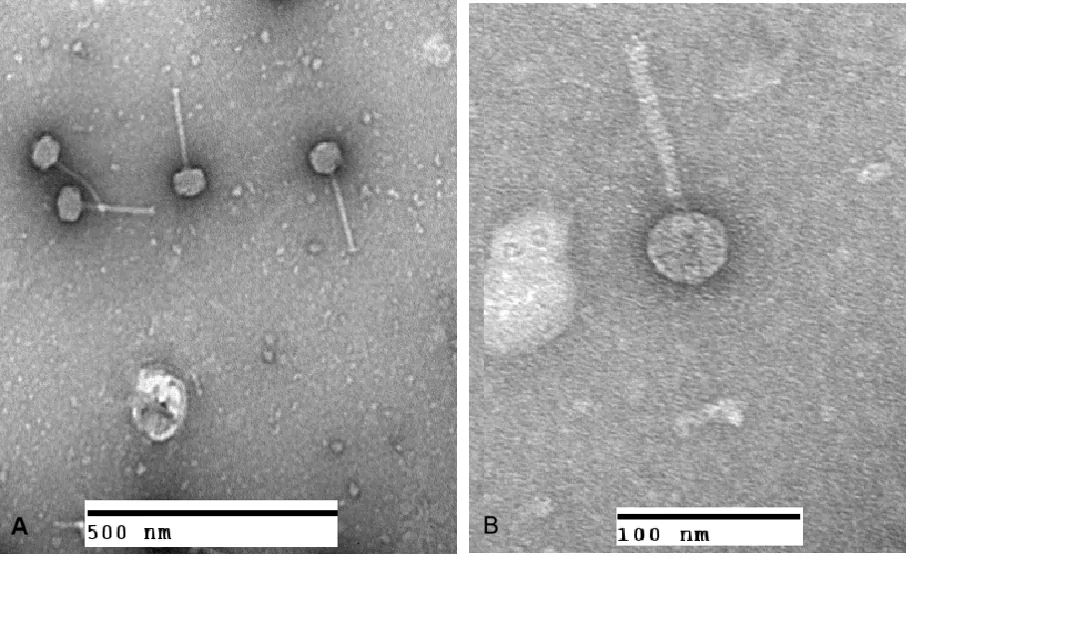



## Description

Bacteriophages are genetically diverse biological entities that can efficiently lyse bacteria and are viewed as a means for controlling bacterial growth via phage therapy (Hatfull, 2020). The discovery and characterization of novel bacteriophages continue to contribute to our understanding of their diversity and complexity. Phages Pulchra and Vanisius were isolated from soil samples using the bacterium *Microbacterium foliorum* NRRL B-24224. *M. foliorum* is a non-pathogenic, gram-positive, rod-shaped bacterium that grows well at 28-30 ˚C. The soil samples were collected in Mount Pleasant (35.4736 N, 87.2497 W) and Franklin (35.918 N, 86.8 W), Tennessee, under the ambient temperature of 28 °C and 31 °C, respectively. Following an enriched isolation protocol, the soil samples were washed in PYCa (peptone-yeast-calcium) medium, the wash was filtered (0.22 μm pore size), and the filtrates were inoculated with *M. foliorum* and incubated at 30˚C with shaking (Zorawik, 2024). After 72 hours of incubation, the cultures were filtered and the filtrates plated in top agar supplemented with *M. foliorum* , incubated at 30 ˚C. Plaques for each sample were purified through 3 rounds of plating, after which lysates were prepared for each phage.

After incubation for 48 hours, the respective phages consistently formed clear plaques on the PYCa agar plates with *M. foliorum* ranging in size from 1 to 2 mm for Pulchra, and from 2 to 3 mm for Vanisius. The number of individual phage plaques used to determine the plaque size ranged between 100-150 plaques per plate. To determine phage morphology using transmission electron microscopy (TEM), the concentrated phage samples were placed on a copper grid and negative stained with uranyl acetate. TEM imaging revealed that Pulchra and Vanisius have a siphovirus morphology, as shown in Figure 1, A and B. The size of phage Pulchra capsid was calculated at 66-68 nm and tail at 143-145 nm (n=4). The phage Vanisius capsid was measured at 44-46 nm and the tail was measured at 104-106 nm (n=4).

Genomic DNA for each phage was isolated from a high-titer phage lysate (~10 ^-10^ pfu/mL) and purified using the Promega Wizard DNA Clean-Up Kit. A sequencing DNA library was prepared using the NEBNext UltraII Library Kit. The genomes were sequenced at the Pittsburgh Bacteriophage Institute on an Illumina MiSeq instrument (v3 reagents) yielding 14,330 single-end 150 bp reads and 38-fold genome coverage for Pulchra, and 301,218-base 150 bp single-end reads and 2467-fold coverage for Vanisius. Raw reads were assembled with Newbler v.2.9 (Russell DA, 2018), and the resulting contigs were checked for completeness by Consed v.29 (Gordon D, 1998). The genomic termini were verified as described (Russell, 2018). Pulchra’s 133,228 bp genome is circularly permuted with 68.8% GC content, and Vanisius’ 17,453 bp genome has 3' single-stranded overhang of 9 CCCGCCCCA bases, and 68.8% GC content.

The genome sequences were annotated using DNA Master v.5.23.6 (Pope & Jacobs-Sera, 2018), embedded with Glimmer v.3.02 (Delcher, 1999) and GeneMark v.2.5p (Besemer & Borodovsky, 2005), PhagesDB BLAST (Altschul, 1990) against the Actinobacteriophage and NCBI nonredundant databases, HHPred v.3.2 (Söding, 2005) against the PDB_mmCIF70, Pfam-v.36, NCBI Conserved Domains databases, Phamerator v.393.0 (Cresawn, 2011), tRNAscanSE v.2.0 (Lowe & Chan, 2016), Aragorn v.1.2.41 (Laslett & Canback, 2004), and PECAAN (http://pecaan.kbrinsgd.org/), all using default software settings.

Pulchra is predicted to encode a total of 92 genes. Based on the gene content similarity (GCS) of at least 35% to actinobacteriophages, Pulchra was assigned to cluster EC (Russell & Hatfull, 2017, Pope, 2017), with which it shares a majority of cluster EC hallmarks. This includes all predicted genes being transcribed unidirectionally, with structure and assembly functions encoded at one end of the genome, lysin A encoded in the middle, and DNA metabolism functions encoded at the other end. No tRNAs or lysogeny-related functions were identified in the genome. Vanisius is predicted to encode 25 genes and is assigned to cluster EE. As with other cluster EE phages, the majority of predicted genes are transcribed unidirectionally and encode putative functions related to virion structure and assembly, with the exception of a few genes that are transcribed in the opposite direction and encode DNA binding proteins. No integrase or immunity repressor functions could be identified in Vanisius or other cluster EE phages, suggesting they are unlikely to establish lysogeny. No tRNA was identified for Vanisius, though a tRNA has been identified in 4 out of the 141 phages of cluster EE, to date.

**Data availability.** Phage Pulchra is available at GenBank with Accession No. MW601217 and Sequence Read Archive (SRA) No. SRX11158998 . Phage Vanisius is available at GenBank Accession No. MN329679 and Sequence Read Archive (SRA) No. SRX11158999 .
